# The impact of a preventive and curative oral healthcare program on the prevalence and incidence of oral health problems in nursing home residents

**DOI:** 10.1371/journal.pone.0198910

**Published:** 2018-06-12

**Authors:** Barbara Janssens, Jacques Vanobbergen, Mirko Petrovic, Wolfgang Jacquet, Jos MGA Schols, Luc De Visschere

**Affiliations:** 1 Community Dentistry and Oral Public Health, Dental School, Ghent University, Ghent, Belgium; 2 Department of Geriatrics, Ghent University Hospital, C.Heymanslaan 10, Ghent, Belgium; 3 Department of Oral Health Sciences ORHE, Faculty of Medicine and Pharmacy, Vrije Universiteit Brussel, Brussel, Belgium; 4 Department of Educational Science EDWE-LOCI, Faculty of Psychology and Educational Sciences, Vrije Universiteit Brussel, Brussel, Belgium; 5 CAPHRI, Dept. Family Medicine and Dept. Health Services Research, Maastricht University, Maastricht, The Netherlands; University of Queensland, AUSTRALIA

## Abstract

**Aims:**

To assess the impact of an oral healthcare program in nursing homes on the initial treatment backlog and residents’ oral health stability.

**Materials and methods:**

The study is a longitudinal cohort study in nursing home residents in Flanders, Belgium, to evaluate the oral healthcare programme Gerodent. The program consisted of: (1) the introduction of an oral healthcare team, (2) oral health education, (3) the implementation of oral health guidelines and protocols, and (4) regular visits of a mobile dental team. Data were extracted from the oral health records of 381 residents from 21 nursing homes who received treatments from the mobile dental team between October 2010 and March 2014 (mean follow-up period of 22.5 months). Oral health and treatment need between baseline and follow-up were compared.

**Results:**

The mean age at baseline was 82.4 years and the mean number of consultations per resident was 3.61 during the follow-up period. The proportion of residents with an oral treatment need was reduced from 65.9% to 31.3%. Among residents with natural teeth, there was significantly lower prevalence of caries (from 70.5% at baseline to 36.5% at follow-up; p<0.001), residual roots (from 54.2% to 25.1%; p<0.001), and need for fillings (from 31.9% to 17.1%; p<0.001) or extractions (from 64.3% to 31.6%; p<0.001). In the group with partial or full dentures (n = 223), 38.1% needed a repair, rebasing or renewal of their existing dentures at baseline and the respective figure at follow-up was 9.0% (p<0.001). In terms of oral health stability, 53% of the residents had no incident restorative and prosthetic treatment need throughout the follow-up period. A lower number of natural teeth at baseline (p<0.001) and a shorter follow-up period (p = 0.002) were associated with higher chances of oral health stability.

**Conclusion:**

The oral healthcare program Gerodent significantly reduced the treatment backlog and contributed to a considerable proportion of residents being stable in terms of oral health without any incident treatment needs.

## Introduction

Poor oral health and a high need for treatment are widespread among nursing home residents. [1–7] This situation is mainly the result of many well-reported barriers concerning oral healthcare experienced by dental professionals, nursing home residents and their (informal) caregivers. [8–14] It is likely to become more pressing in the future, as a growing number of remaining natural teeth and complex prosthetic rehabilitations will create the need for increasingly complex oral healthcare. [6]

To meet these barriers and this need for more complex treatments, mobile and portable dentistry at nursing homes have been suggested as a safe, cost-effective alternative for regular dental clinics. [15–18] Nevertheless, there is little information on the implementation of these on-site services. One short regional Austrian report concluded that mobile dentists were active in 51.5% of the nursing homes, but these mobile services were not embedded in a structured program. [19] In contrast, in other countries such as Sweden, a structured program is already in place at a national level. [20]

Although a comprehensive system is often still lacking, general guidelines for mobile and on-site dental care have already been formulated by Helgeson et al. [16]:

“These delivery systems are not simply traditional dental practices located in nursing homes. They are interdisciplinary team efforts designed to address the oral health needs of nursing home residents systematically. The provision of dental care involves not only dental staff, but also nursing staff, primary care physicians, resident representatives, and third-party payers, each of whom has an important role to play. In addition, on-site delivery systems must assist in establishing preventive programs, provide education for nursing staff, and participate actively in the medical-dental management of medically compromised patients.”

These guidelines have already been applied in some mobile dental clinics [21,22] and have been shown to achieve oral health stability, requiring only diagnostic or preventive services at periodic examination, in up to 44% of the residents. [23] The success of the approach has also been illustrated in a study by Sjögren et al., which demonstrated that professional domiciliary care, limited to professional cleaning, reduced dental plaque and gingivitis in nursing home residents. [24] Nevertheless, there remains an overall paucity of information on how the implementation of an oral healthcare strategy including a mobile dental team for preventive and curative treatment impacts on the oral health of nursing home residents.

In two provinces of Flanders, Belgium (i.e. East and West Flanders), a preventive and curative oral healthcare program for nursing homes called ‘Gerodent’ was introduced in 2010. In line with the guidelines for mobile dental care [16], this program comprises a preventive protocol at the nursing home level, education for caregivers and preventive and curative care for residents. Previous research on this oral healthcare program for nursing homes revealed that (1) the nursing home residents’ oral health was compromised [2], (2) the residents’ high intake of hyposalivation-related medication affected their oral health [25], (3) the preventive part of the program increased the care staffs’ oral health-related knowledge [26], and (4) the provision of preventive and curative on-site dental care had an additional positive effect on the care staffs’ oral health-related knowledge and attitude. [27]

The aim of the present study was to assess how a preventive and curative oral healthcare program like Gerodent may impact on the initial treatment backlog in nursing homes and how it may affect residents’ oral health stability.

## Materials and methods

### Study design

The present study is a longitudinal cohort study in nursing home residents in Flanders. It was approved by the Ethical Committee of Ghent University Hospital (B670201318461) and written informed consent was obtained from all nursing homes. Moreover, written consent from the individual participants in the study was not required since the study was based on a retrospective data analysis of existing patient records in a strictly anonymous way. At the time of data analyses most of the participants had died given that the mean survival time in a nursing home is little more than one year. The data have been analyzed two years after the latest information gathered in the study.

### Study population, study sample and study settings

The study population consisted of nursing home residents in East and West Flanders (i.e. two Belgian provinces) with difficult access to regular oral healthcare due to physical and/or cognitive impairment. The study sample was derived from a previous sample containing residents of 23 nursing homes in the Gerodent oral healthcare network. This sample consisted of 1,226 residents who visited the mobile dental clinic for a first consultation between October 2010 (i.e. when Gerodent started) and April 2012. The socio-demographic characteristics and baseline oral health status of this sample were described in a previous study conducted by the same authors. [2] To be included into the subsample of the present study, the residents needed to have follow-up data in their oral health records for a minimum of 11 months after the first screening.

### Exposure

The exposure in this study was Gerodent, a preventive and curative oral healthcare program for nursing homes. This The program involves (1) the introduction of an oral healthcare team in the nursing home, consisting of one nursing home project supervisor, at least two oral healthcare organizers (nurses or nurses’ aides) per ward, a physician, and possibly an occupational or speech therapist; (2) oral health education for the managing director and for the nursing staff; (3) the implementation of the guideline "Oral healthcare Guideline for Older people in Long-term care Institutions" (OGOLI) and the daily oral healthcare protocol derived from this guideline [28–32]; and (4) regular visits of a mobile dental team to support the nursing staff and deliver preventive and curative oral healthcare to residents who cannot access regular dental care. The details of the oral healthcare program have been described in previous articles and will therefore not be repeated here. [26,27]

### Data collection

Data were extracted from the oral health records of the nursing home residents receiving oral treatments between October 2010 and March 2014 (study period) from the Gerodent mobile dental team. These records include an oral, medical, physical and cognitive assessment. The latter three were performed by the caring staff and physician of the nursing home; the oral assessment was performed by the three dentists of the Gerodent team (first, second and last author), who are all experienced in geriatric dentistry. For the registration of the oral health status, the dentists had a fully equipped mobile dental unit at their disposal with a portable dental operating light (Aseptico). A mobile x-ray device (Rextar EXO1414) was available to ensure a correct diagnosis and draft the most suitable treatment plan. The data considered for this study included (1) demographic variables (i.e. age, gender, nursing home of residence, care dependency and the right to increased reimbursement), (2) medication intake (i.e. the number of (hyposalivation-related) medications), (3) the oral health status (i.e. the number of natural teeth, residual roots, filled teeth, decayed teeth, D_3_MFt being the sum of teeth with visually obvious dental decay in the dentine of the tooth (D_3_), missing teeth (M) and filled teeth (F), the presence of implants and removable dentures), (3) an assessment of the treatment need (i.e. the need for fillings and extractions, treatment index, restorative index and the need for rebasing, repair or renewal of dentures), and (4) all the oral treatments provided during the follow-up period. The above-mentioned variables were extensively described in previous studies. [2,25] The baseline data were collected during the first consultation (T_0_), the final data collection took place during the last consultation within the study period (T_1_). The length of the follow-up period was determined by the time between T_0_ and T_1_, while care dependency was based on the Katz index of Independence in Activities of Daily Living including Activities of Daily Living (ADL) and cognition. Increased reimbursement, mentioned in (1), is a governmental measure for people who are entitled to a higher reimbursement for healthcare provision due to their low income. More information on the included data can be found in previous articles. [2,25]

### Outcome and explanatory variables

The outcome variables were the oral health status and treatment need at baseline and at the end of the follow-up period, the extent to which the treatment backlog was eliminated, and the proportion of residents with oral health stability during the follow-up period. The elimination of the treatment backlog was expressed as a reduction in the number and percentage of residents with a need for oral treatment (i.e. a need for fillings, extraction, repair, rebasing or renewal of dentures) from baseline to follow-up. Oral health stability was interpreted as a situation in which no new dental (fillings or extractions) or prosthetic (repair, rebasing, renewal) treatment was needed until the end of the follow-up period.

To assess which factors could affect the elimination of the treatment backlog and the incidence of new oral health problems, the following explanatory variables were registered: the residents’ age, gender, care dependency, increased reimbursement for health costs, number of medications, number of natural teeth at baseline, any presence of a (partial or full) removable denture, and the duration of the follow-up period. More information on the explanatory variables can be found in previous articles. [2,25]

### Statistical analysis

Descriptive analyses were performed of all socio-demographic variables and variables expressing the oral health status or the treatment need at baseline and at the end of the follow-up period. To explore the differences between the oral health status and treatment needs at baseline (T_0_) and after follow-up (T_1_), the non-parametric Wilcoxon matched pairs signed ranks test and McNemar test were used. A logistic regression analysis was performed to assess the impact of the explanatory variables on the treatment backlog elimination and the oral health stability. Tests resulting in *p*-values < 0.05 were considered significant. All analyses were performed with SPSS for Windows version 22 (SPSS Inc., Chicago, IL, USA).

## Results

The study sample consisted of 381 residents from 21 different nursing homes with a mean follow-up period in the oral healthcare program of 22.5 months (SD 7.3, range 11.0–40.1). The mean age at baseline was 82.4 years (SD 8.9, range 30–100) and the sample was mainly female (n = 275, 72.2%). The mean number of medications was 9.08 (SD 3.4, range 0–22), of which 4.69 (SD 2.1, range 0–12) could induce a dry mouth (Table 1). Considering the preventive and curative treatment, the mean number of consultations per resident was 3.61 (median 3.00, SD 3.03, range 1–19) during the follow-up period.

**Table 1:** Baseline socio-demographic data of the participants (total n = 381).

Variable	Mean (median) or Number	SD or %
**Age (years)**		
Total sample	82.4 (83.8)	8.9
< 65	15	3.9%
65–79	92	24.1%
80–89	182	47.8%
> 89	92	24.1%
**Gender**		
Male	106	27.8%
Female	275	72.2%
**Increased reimbursement**	253	66.4%
**Care dependency**		
Low (Katz O and A)	75	19.7%
Medium (Katz B)	109	28.7%
High (Katz C and Cd)	196	51.6%
**Number of medications**	9.1 (9.0)	3.4
**Number of hyposalivation-related medications**	4.7 (5.0)	2.2

If the residents’ oral health status at baseline is compared with their oral health status at the end of the follow-up period, it becomes clear that the oral healthcare program resulted in fewer natural teeth and more full dentures (Table 2).

**Table 2:** Residents’ general oral health status (total n = 381).

Variable	Baseline (T_0_)		Follow-up (T_1_)	
	Number or Mean (median)	SD or %	Number or Mean (median)	SD or %
**Residents with natural teeth**	263	69%	234	61%
**Number of natural teeth**				
Total Sample	9.0 (7.0)	8.8	7.0 (4.0)	8.8
0 teeth	118	31.0%	147	38.6%
1–9 teeth	105	27.6%	112	29.4%
10–20 teeth	107	28.1%	90	23.6%
> 20 teeth	51	13.4%	32	8.4%
**D**_**3**_**MFt** ^**a**^	26.2 (29.0)	6.8	27.2 (31.0)	6.6
**Implants**	3	0.8%	8	2.1%
**Full denture upper and lower jaw**	105	27.6%	113	29.7%
**Overdenture upper or lower jaw**	10	2.6%	11	2.9%
**Full denture upper jaw in combination with natural teeth (and partial denture) lower jaw**	66	17.3%	61	16.0%
**Full denture lower jaw in combination with natural teeth (and partial denture) upper jaw**	12	3.1%	6	1.6%
**Natural teeth in combination with partial denture**	40	10.5%	37	9.7%

^a^ D_3_MFT: sum of teeth with obvious dental decay in the dentine of the tooth D_3_, missing teeth M and filled teeth F

If only considering the residents with natural teeth (n = 263), the mean D_3_MFt increased significantly from 23.60 to 25.15 (*p* < 0.001) during the follow-up period. A significant decrease in oral pathology could be observed, as expressed by the number of decayed teeth (*p* < 0.001) and residual roots (*p* < 0.001). As a consequence, the oral healthcare program resulted in an increased treatment and restorative index (from 87.24% to 94.09%, p < 0.001 and from 31.63% to 64.19%, *p* < 0.001 respectively). The need for treatment at the level of natural dentition fell from 3.76 to 1.31 teeth to be filled or extracted (*p* < 0.001) (Table 3). Approaching these numbers in a dichotomous way, the oral healthcare program reduced the proportion of residents with caries (from 70.5% to 36.5%, *p* < 0.001), residual roots (from 54.2% to 25.1%, *p* < 0.001), and a need for fillings (from 31.9% to 17.1%, *p* < 0.001) or extractions (from 64.3% to 31.6%, *p* < 0.001). Simultaneously, the proportion of residents with a treatment or restorative index of 100% rose from 29.1% to 63.5% (*p* < 0.001) and from 14.7% to 49.5% (*p* < 0.001), respectively.

**Table 3:** Oral health status and treatment need of residents with natural teeth at baseline (total n = 263).

Variable	Baseline (T_0_)		Follow-up (T_1_)		*p*-value^a^
	Mean (median) or Number	SD or %	Mean (median) or Number	SD or %	
**Number of natural teeth**					
Total Sample	13.09 (13.0)	7.64	10.16 (9.0)	7.92	< 0.001
0 teeth	0	0	29	11.0	
1–9 teeth	105	39.9	112	42.6	
10–20 teeth	107	40.7	90	34.2	
> 20 teeth	51	19.4	32	12.2	
**D**_**3**_**MFt**	23.60 (25.00)	6.70	25.15 (27.00)	6.71	
Decayed teeth	3.02 (2.00)	4.01	1.40 (2.95)	2.95	< 0.001
Missing teeth	18.90(19.00)	7.64	21.86 (23.0)	7.89	< 0.001
Filled teeth	1.62 (0.00)	2.72	1.89 (1.00)	2.55	0.003
**Number of residual roots**	1.83 (1.00)	3.35	0.85 (0.00)	2.37	< 0.001
**Treatment index** ^**b**^	87.24 (92.59)	15.71	94.09 (100.00)	12.07	< 0.001
**Restorative index** ^**c**^	31.63 (0.00)	38.54	64.19 (89.20)	41.73	< 0.001
**Fillings needed**	0.81 (0.00)	1.53	0.37 (0.00)	1.03	< 0.001
**Extractions needed**	2.95 (1.00)	4.31	1.31 (0.00)	3.30	< 0.001
**Total treatment need (fillings + extractions)**					
Total Sample	3.76 (2.00)	4.44	1.68 (0.00)	3.50	< 0.001
1–9 teeth	2.41 (2.00)	2.33	1.04 (0.00)	1.77	<0.001
10–20 teeth	4.21 (3.00)	4.50	2.48 (0.00)	4.35	<0.001
> 20 teeth	5.61 (3.00)	6.44	3.19 (1.00)	5.43	0.016

^a^ Wilcoxon matched pairs signed ranks test

^b^ The treatment index is derived from the DMFt index and expresses the percentage of decayed teeth that received restorative treatment or were extracted. It is calculated by the following formula: [(F+M)/(D+F+M)] x 100. The more untreated caries, the lower the restorative index. The treatment index is especially relevant compared to the restorative index when the number of missing teeth is high.

^c^ The restorative index is derived from the DMFt index and expresses the percentage of decayed teeth that received restorative treatment. It is calculated by the following formula: [F/(D+F)] x 100. The more untreated caries, the lower the restorative index.

During the follow-up period, 79.1% of the residents with natural teeth received fillings and/or extractions with a mean number of 3.86 treated teeth per person (SD 4.07). A considerable part of the residents experienced new pathology during the follow-up period, resulting in 53.2% of the residents requiring new fillings or extractions. The mean number of teeth with pathology occurring during the follow-up period was 1.78 (SD 2.84; Table 4).

**Table 4:** Dental treatment and new pathology in residents with natural teeth at baseline (n = 261) during the follow-up period (T0—T1).

Variable	n	%	Mean (median)	SD
**Fillings**	111	42.2	0.98 (0.00)	1.56
**Extractions**	173	65.8	2.90 (1.00)	3.70
**Total treatment (fillings + extractions)**	208	79.1	3.86 (2.00)	4.07
**New fillings needed during follow up period**	86	32.7	0.69 (0.00)	1.34
**New extractions needed during follow up period**	92	35.0	1.08 (0.00)	2.50
**Total new treatment need (fillings + extractions)**	140	53.2	1.78 (1.00)	2.84

In the group of residents with partial or full dentures (n = 223), a major treatment backlog was also observed at baseline: 85 residents (38.1%) needed a repair, rebasing or renewal of their existing dentures. This treatment backlog was reduced to 20 residents (9.0%) after the follow-up period (*p* < 0.001; Table 5). During the follow-up period, 39 residents (17.5%) received a repair, 63 (28.3%) a rebasing and 21 (9.4%) a renewal of the existing dentures at baseline. In total, 103 residents (46.2%) received some kind of prosthetic treatment.

**Table 5:** Treatment need of residents with dentures (total n = 223).

Variable	Baseline (T_0_)		Follow-up (T_1_)		*p*-value^a^
	Number	%	Number	%	
**Need for repair**	32	14.3	14	6.3	0.006
**Need for rebasing**	54	24.2	3	1.3	< 0.001
**Need for renewal dentures**	14	6.3	3	1.3	0.013
**Overall treatment need dentures**	85	38.1	20	9.0	< 0.001

^a^McNemar test

All the above-mentioned data allow us to assess the extent to which the initial treatment backlog was eliminated and evaluate the oral health stability during the follow-up period. At baseline, there was a treatment backlog for 251 residents (65.9%), which was reduced to 120 residents (31.3%) after the follow-up period. In the group of residents without a treatment need at baseline (n = 130), 13.1% (n = 17) had a treatment need after follow-up. In the group of residents with a treatment need at baseline (n = 251), 40.8% (n = 102) still had a treatment need at follow-up. No less than 204 residents (53.5%) achieved oral health stability during the follow-up period, meaning that there were no new natural teeth with a need for treatment and there was no need for new prosthetic treatment. In the group with baseline treatment needs (n = 251), 50.5% (n = 126) remained stable over time compared to the group without baseline treatment needs (n = 130) where 60.0% (n = 78) remained oral health stability.

To assess the impact of the explanatory variables on the treatment backlog and the oral health stability at the end of the follow-up period (T_1_), a logistic regression analysis was performed. A higher number of natural teeth was associated with higher chances of having a treatment need at T_1_ (*p* < 0.001) and for not achieving oral health stability (*p* < 0.001). The duration of the follow-up period was also a predicting variable for oral health stability: A longer follow-up period was associated with lower chances of achieving oral health stability (*p* < 0.002; Tables 6 and 7).

**Table 6:** Logistic regression analysis for the treatment need at the end of the follow-up period (T_1_).

Variables (reference)		Est β	*p*-value	OR	95% C.I.	
					Lower	Upper
**Age**		0,027	0,078	1,027	0,997	1,058
**Gender (Female)**	Male	0,207	0,464	1,230	0,707	2,141
**Care dependency (Low)**	Medium	0,253	0,519	1,288	0,597	2,782
	High	0,411	0,250	1,509	0,749	3,041
**Increased reimbursement (Yes)**	No	0,499	0,055	1,646	0,990	2,739
**Number of medications at baseline**		-0,024	0,521	0,976	0,907	1,050
**Number of natural teeth at baseline**		0,117	**< 0,001**	1,125	1,080	1,171
**(Partial) denture at baseline**		-0,319	0,363	0,727	0,365	1,446
**Duration of the follow-up period**		0,008	0,621	1,008	0,975	1,042

**Table 7:** Logistic regression analysis for the oral health stability at the end of the follow-up period (T_0_ -T_1_).

Variables (reference)		Est β	*p*-value	OR	95% C.I.	
					Lower	Upper
**Age**		0,002	0,905	1,002	0,974	1,030
**Gender (Female)**	Male	-0,087	0,749	0,917	0,539	1,560
**Care dependency (Low)**	Medium	-0,380	0,257	0,684	0,355	1,319
	High	0,144	0,645	1,154	0,627	2,124
**Increased reimbursement (Yes)**	No	0,389	0,115	1,475	0,910	2,391
**Number of medications**		-0,058	0,102	0,944	0,880	1,011
**Number of natural teeth**		-0,091	**< 0,001**	0,913	0,880	0,948
**Prosthetic treatment need at baseline**		-0,015	0,964	0,985	0,521	1,865
**Duration of the follow-up period**		-0,049	**0,002**	0,952	0,923	0,983

## Discussion

Untreated tooth decay in permanent teeth remains the most prevalent health condition across the globe, with a prevalence of 35.8% in Western Europe and an incidence of 49,344 per 100,000 person years in 2010. [33] In our sample, a high rate of untreated oral pathology could be observed at baseline, with 70.5% of the residents suffering from untreated caries. This high prevalence of untreated caries, also called Frail Elder Caries (FEC) is a serious threat to the overall health and well-being of nursing home residents. [34] The oral healthcare program Gerodent was able to reduce this percentage to 36.5%, a proportion similar to the mean prevalence in Western Europe.

The need for treatment observed in 65.9% of the residents at baseline was halved to 31.3% by the end of the study period. Similarly, Gerritsen et al. registered a treatment need in 44.4% of the residents in a nursing home with integrated dental care compared to 86.9% of the residents in a nursing home with incidental dental care. [35] In contrast, another study by Gerritsen et al., measuring the dental treatment need of 432 residents of nursing homes with integrated oral healthcare, reported 72% of the residents requiring oral treatment. These differences in results can possibly be explained by different interpretations of integrated oral healthcare. In the present study, the need for treatment was reduced by means of basic curative oral healthcare including fillings, extractions and prosthetic treatment. As has been shown by Morgan et al., these basic interventions suffice to eliminate most of the dental treatment needs among nursing home residents. [36] In the future, more complex treatments may need to be provided due to the increased complexity of oral health status in general.

In addition to a reduced need for treatment, the program provided oral health stability for a considerable part of the sample (53.5%). A study by Smidt et al., measuring oral health stability for 24 months in 868 nursing home residents from 62 nursing homes, examined the effects of a similar oral healthcare program and observed that 44% of the residents achieved oral health stability during their participation in the program. [23] Although this is less than the percentage obtained in the present study, these results may be explained by the specific study design, as one of the inclusion criteria was the presence of at least one natural tooth. If we apply this inclusion criterion to the sample of the present study, the oral health stability becomes 43% and thus equal to the study of Smidt et al. Another interesting finding from Smidt et al. is that predicting variables for achieving oral health stability were being relatively younger, female, residing in proprietary homes and initially exhibiting a low need for treatment. In the present study the initial treatment need was strongly correlated with the number of natural teeth so only one of these variables was included into the logistic regression. Besides the initial treatment need, the other findings of Smidt et al. are not confirmed in the present study, so more research is needed to clarify these inconsistencies.

Previous research showed that persons with cognitive impairment have worse oral health compared to persons without cognitive impairment but also that oral care capacity mediates the association between cognition and dental caries severity in older adults. [37–39] In the present study, a high care dependency (including cognitive impairment) didn’t result in a higher risk of oral health instability. As a consequence of the preventive protocols and the education of the caregivers, the residents with low oral care capacities probably received support with their daily oral hygiene resulting in the elimination of oral health inequalities based on care dependency.

A study of Chalmers et al., observing the caries incidence and increments in dentate nursing home residents in a one-year period, concluded that 72.1% of the residents showed caries increments between baseline and follow-up. [40] Coronal caries incidence was 64.4% and root caries incidence 48.5%. This was still an underestimation because some surfaces could not be assessed due to the high plaque levels. In the present study, 53.2% of the dentate residents obtained a new need for dental treatment (i.e. filings or extractions). This proportion is lower than in the study of Chalmers et al. and might be attributed to the preventive aspects of the oral healthcare program. Nevertheless, 53.2% is still a considerable percentage. During the study period, the residents used 1450 ppm fluoridated toothpaste without any additional fluoride applications. However, the literature recommends 5000 ppm fluoridated toothpaste or the regular application fluoride varnishes for frail older people. [41,42] Applying these recommendations could further improve the outcomes of the oral healthcare program.

### Limitations

The design of the present study did not allow us to compare the results with a control group that did not participate in the oral healthcare program. However, not providing dental treatment to a control group during a mean period of 22 months in a study sample of frail older nursing home residents might have raised ethical considerations due to the high mortality rates in nursing homes.

Changes in care dependency during the study period were not taken into account. At baseline, 51.6% of the residents in the sample had the highest possible level of care dependency so they remained in the same category along the follow-up period. However, resident belonging to the low or medium care dependency group might have evolved to a higher care dependency along the follow-up period. Nevertheless, their care dependency will have been lower for a certain period of time so the “mean value” for their care dependency will always be lower compared to the group with higher care dependency.

Furthermore, for feasibility reasons, no plaque measurements were performed in this study. The changes in oral hygiene levels could have provided more insight into the incidence of caries during the follow-up period. Moreover, co-morbidity and nutritional intake were not taken into account as an explanatory variable.

## Conclusion

The oral healthcare program Gerodent significantly reduced treatment backlog and contributed to a considerable proportion of residents being stable in terms of oral health without any incident treatment needs.
